# An unfavorable biologic profile associated with decreased overall survival and cancer-specific survival in non-metastatic breast cancer: A latent class analysis

**DOI:** 10.1016/j.tranon.2026.102694

**Published:** 2026-02-13

**Authors:** Claire Falandry, Sigrid Hatse, Barbara Brouwers, Cindy Kenis, Ann Smeets, Patrick Neven, Charlotte Cuerq, Frederic Pamoukdjian, Karim Chikh, Hans Wildiers

**Affiliations:** a Laboratoire CarMeN, INSERM U1060/Université Lyon 1/INRAE U1397/Hospices Civils Lyon, Pierre-Bénite, France; b Service de Gériatrie, Centre Hospitalier de la Croix-Rousse, Institut du Vieillissement, Hospices Civils de Lyon, Lyon, France; c Laboratory of Experimental Oncology, Department of Oncology, KU Leuven, Leuven, Belgium; d Department of General Medical Oncology, UZ University Hospitals Leuven, Leuven, Belgium; e Department of Geriatric Medicine, University Hospitals Leuven, Leuven, Belgium; f Department of Public Health and Primary Care, Academic Centre for Nursing and Midwifery, KU Leuven, Leuven, Belgium; g Department of Surgical Oncology, University Hospitals Leuven, Leuven, Belgium; h Department of Gynecology and Obstetrics, University Hospitals Leuven, Leuven, Belgium; i Biochemistry Department, Centre Hospitalier Lyon Sud, Hospices Civils de Lyon, Pierre-Benite, France; j Service de Médecine Gériatrique, Hôpital Avicenne, APHP, Bobigny, France; k Inserm UMR_S942, Cardiovascular Markers in Stressed Conditions, MASCOT, Université Sorbonne Paris Nord, Bobigny, France; l Multidisciplinary Breast Center, University Hospitals Leuven, Leuven, Belgium

**Keywords:** Aging biomarkers, Inflammaging, Telomere dysfunction, Breast cancer, Geriatric oncology

## Abstract

**Background:**

Chronological age is an imperfect proxy for risk assessment in geriatric oncology. There is an urgent need for an objective, easily measurable biological aging signature to refine patient stratification and personalize therapeutic decisions.

**Methods:**

We analyzed a panel of seven aging-related biomarkers (including markers of inflammation, anabolic reserve, and telomere status) in 244 nonmetastatic breast cancer patients from two age groups (“Old”, ≥70 years, *N* = 162; “Young”, ≤60 years, *N* = 82). We used Latent Class Analysis (LCA) to integrate these markers and identify distinct biological risk profiles. These profiles were then evaluated for their association with Overall Survival (OS) and Cancer-Specific Death (CSD) via Competing Risk Analysis.

**Results:**

LCA identified two patient profiles. The Unfavorable Biologic Profile (56.1% of the cohort) was defined by a triad of high MCP-1, high Chitinase activity, and low IGF-1. This profile was strongly associated with poorer OS (Age-adjusted HR=1.82, *p* = 0.018). Crucially, 15% of chronologically “Young” patients were assigned to this high-risk profile, while 23% of “Old” patients were assigned to the Favorable Profile. Furthermore, the Unfavorable Profile was more strongly and specifically associated with CSD (Subdistribution HR: 2.05, *p* = 0.012) than with Non-Cancer Death.

**Conclusion:**

Our results delineate an unfavorable, trans-chronological biological profile that identifies patients with low host reserve, largely driven by inflammaging and catabolism. This integrated signature provides a robust, objective screening tool to identify biologically frail patients, validating the need for Comprehensive Geriatric Assessment (CGA) and biomarker-guided therapeutic de-escalation (e.g., avoiding adjuvant chemotherapy) to improve individualized outcomes in oncology.Trial registration: BS32220096117.

## Highlights

• Latent Class Analysis (LCA) integrates multiple aging markers (IGF-1, MCP-1, Chitinase) into a single, robust Biological Risk Profile.
• This Unfavorable Biological Profile transcends chronological age, identifying biologically frail patients in both "Old" (≥70) and "Young" (≤60) cohorts.
• The integrated profile is a strong and specific predictor of Cancer-Specific Death, outperforming individual biomarkers and chronological age.
• This measurable aging signature may help clinicians evaluate patients’ individual expectancy to tailor (adjuvant) treatment strategies.

## Introduction

Breast cancer is the most common female cancer, affecting at least 1 in 8 women during their lifetime, and remains the second leading cause of cancer mortality in women worldwide [1]. Approximately 30 % of cancer diagnoses and 50 % of cancer-related deaths involve patients aged ≥70 years, highlighting the critical need for individualized treatment strategies in this population [2]. Given the overall reduced benefit and increased toxicity associated with adjuvant chemotherapy in older adults, particularly in the context of luminal cancers [3,4]. the decision to administer or withhold treatment relies heavily on an accurate, individualized estimation of the patients’ residual life expectancy [5].

Current tools, such as chronological age and comprehensive geriatric assessment (CGA), often fail to capture the vast heterogeneity of biological aging at the individual level, leading to potential overtreatment in some patients and undertreatment in others. While complex multiomics and large panels have been used to develop intricate "aging clocks," these approaches lack the feasibility and direct transferability required for routine clinical oncology practice [6]. There is an urgent need for easily measurable, blood-based biomarkers that reflect pathological aging, frailty, and healthy longevity to improve prognostic stratification beyond conventional metrics.

To address this unmet need, we focused on a curated panel of general biological aging biomarkers, selected for their strong mechanistic link to the core Hallmarks of Aging and cancer progression. This panel includes markers of chronic inflammation (IL6, MCP-1, RANTES), nutrient sensing (IGF-1), and telomere dysfunction (T/S ratio, CRAMP/LL-37, and Chitinase 1) [[7], [8], [9], [10], [11]]. We chose this comprehensive, multi-domain panel because no single "perfect aging biomarker" exists, and an integrated aging profile is likely best captured by exploring multiple distinct biological pathways.

Crucially, our study used the breast cancer setting—the most common malignancy in women—as an oncologically well-defined and frequently occurring model to test the relevance of general aging biomarkers in clinical decision-making. The markers assessed are drivers of aging and not specific prognostic markers of the tumor itself. These drivers of aging are interconnected, suggesting that integrated statistical approaches are required to delineate meaningful prognostic patient subgroups, or "risk phenotypes."

Latent Class Analysis (LCA) is a powerful, integrated statistical strategy used to uncover hidden clusters in data. Given the clinical imperative to identify patients crossing pathological thresholds (e.g., high inflammation or severe telomere dysfunction), we applied the LCA framework to the binarized biomarker data. This methodology yields distinct, clinically interpretable classes based on the simultaneous presence of multiple high-risk states [12].

The present analysis was performed on a cohort from the Leuven Multidisciplinary Breast Center (LMBC, UZ Leuven, Belgium) biobank [13], enriched with the selected aging biomarkers since 2003 [14,15]. The identification of distinct latent biological profiles strongly associated with adverse outcomes raises a fundamental biological question regarding the interplay between the host and the malignancy: is a poor outcome driven by a systemic inflammatory "terrain" (or biological age) that precedes the tumor and promotes its aggressiveness, or is the unfavorable biomarker profile a reactive consequence induced by an intrinsically aggressive tumor that drives a severe catabolic and inflammatory host response? Addressing this question requires a specific focus on the cause of death.

The aim of this study was to identify latent classes defined by combinations of aging biomarkers and to evaluate the prognostic association between these resulting patient profiles and overall survival (OS), with a critical focus on the Cancer-Specific Death (CSD) risk using a Competing Risk Analysis (CRA) model, aiming to better distinguish the impact of host heterogeneity on the outcome.

## Materials and methods

### Patient groups

In 2000, Leuven Multidisciplinary Breast Center (LMBC) in UZ Leuven, Belgium, opened a prospective clinical database containing patient and tumor-related information, as well as clinical follow-up information such as treatment, relapse and cause of death of all patients with invasive breast cancer diagnosed and treated at UZ Leuven. Later, prospective blood samples were also collected, including baseline serum, plasma and leukocyte RNA samples from newly diagnosed patients as of 2003 and germline (leukocyte) DNA samples as of 2007 [13]. All clinical data were collected in compliance with the Helsinki Declaration. Blood sampling, collection of patient data, and genetic analysis were approved by the ethics committee of University Hospitals Leuven. All patients included in the study provided written informed consent. Brouwers et al. explored the associations between biomarkers and frailty markers in 244 patients, 162 aged ≥ 70 years (“old” group) and 82 aged ≤ 60 years (“young” group) [14]. Both groups were selected from the LBMC database: the “Old” group corresponded to patients included at the time of diagnosis of early or locally advanced (i.e., nonmetastatic), primary or second primary breast cancer with a geriatric assessment and baseline blood sampling performed before initiation of any chemotherapy or radiotherapy (with neoadjuvant hormonotherapy allowed). The “young” group included patients with similar diagnoses of early or locally advanced breast cancer and with a baseline blood sample available. Both groups were carefully balanced in terms of comparable relative contributions of the different histological subtypes (Luminal A-like, Luminal B-like, Luminal B-HER2 positive, HER2 positive and Triple Negative breast tumors) [16]. For the current study, follow-up data were extracted from the LMBC clinical database with a cutoff date of 10 October 2022 (median follow-up of 128.8 months for the whole group) to perform OS analysis.

### Biomarker analysis

The following cytokine measurements were previously performed on serum samples via ELISA kits according to the manufacturers’ instructions as previously reported [14]. IL6, CCL5/RANTES, CCL2/MCP-1, and IGF-1. The mean leukocyte telomere length was also assessed via a quantitative polymerase chain reaction assay on DNA extracted from EDTA buffy coats as previously reported. Telomere length was expressed as the ratio ‘T/S’ of telomeric DNA relative to the single-copy housekeeping gene 36B4 [14]. In addition, two aging biomarkers identified as being released in the context of telomere dysfunction [11]. were included in the biomarker dataset: chitinase and LL-37, the human homologs of CRAMP (cathelicidin-related antimicrobial peptide). Serum LL-37 levels were measured via an LL-37 ELISA kit (HK321, Hycult Biotech) according to the manufacturer’s instructions. Chitinase activity (chitotriosidase activity) was measured with a chitinase assay kit (CS1030; Sigma‒Aldrich). Each reaction needed 5 µL of serum mixed with substrate solution (4-methylumbelliferone). Enzyme activity was determined via fluorimetry at an excitation wavelength of 360 nm and an emission wavelength of 450 nm.

### Statistical analyses

#### Individual biomarkers: correlation matrix, univariate survival, and linearity assessment

After multivariate imputation by chained equations (MICE package of R), a Spearman correlation matrix between the crude measurement of biomarkers was built. Overall survival (OS) was defined as the time interval between the date of breast cancer diagnosis and the date of death from any cause. Continuous variables were dichotomized based on their median value to define high and low categories for survival analyses. Kaplan-Meier (KM) survival curves were plotted, and the log-rank test was used to compare OS between groups. To control the family-wise error rate due to multiple comparisons performed on the seven aging biomarkers, the P-values resulting from the log-rank tests were adjusted using the False Discovery Rate (FDR) method proposed by Benjamini and Hochberg (BH). The adjusted P-values (q-values) were used to determine the statistical significance of each biomarker's association with overall survival. A two-sided p-value or q-value of <0.05 was considered statistically significant. To justify the use of dichotomized thresholds, we also performed a multivariate logistic regression using the continuous, log10-transformed values of the biomarkers to verify if the association with OS was linear or threshold-dependent.

### Latent class analysis and survival analysis

#### Sequential modeling strategy and validation of clinical feasibility

Our statistical approach is sequential and relies on three distinct steps: (1) Risk Phenotype Identification (using LCA), (2) Prognostic Evaluation (using Cox models and CRA) and (3) Clinical Feasibility Validation (using Logistic Regression and AUC)

**Step 1** **: Risk phenotype identification**

*Rationale for Latent Class Analysis (LCA)*

We used Latent Class Analysis (LCA) to identify multi-biomarker biological profiles (latent classes) from the full panel of 7 biomarkers. Biomarkers were initially transformed into categorical variables (binary: “high” versus “low”) using the median value of the entire cohort as the clinical threshold. This step was performed to capture the synergistic risk associated with crossing key pathological thresholds simultaneously, which is highly relevant in clinical oncology and geriatric risk assessment. Although the biomarkers were continuous variables—for which Latent Profile Analysis (LPA), a continuous finite mixture model, is typically considered—we performed a rigorous methodological investigation to assess the feasibility of both approaches because of two main methodological issues: LPA instability and LCA robustness. An exhaustive search of LPA models (including all configurations from VVV to EII, i.e., models 9, 6, 4, 5, 2, and 1, as defined by the mclust framework and implemented via tidyLPA) was attempted to delineate three latent profiles. None of the LPA models converged due to numerical instability or resulted in unstable solutions with clinically non-viable class sizes (e.g., 1.2 % of the cohort). This failure of continuous finite mixture modeling, often attributed to deviations from multivariate normality (confirmed by Mardia’s test, *p* < 0.001), definitively precluded the use of LPA. Consequently, we adopted Unsupervised Latent Class Analysis (LCA), a method known for its robustness with binarized, threshold-based indicators. This methodology yields distinct, clinically interpretable classes based on the simultaneous presence of multiple high-risk states, aligning with the clinical imperative to identify individuals crossing pathological thresholds [12,17].

*Latent Class Modeling*

The latent class indicators included the binarized values for chitinase, LL-37, IGF-1, IL6, MCP-1, RANTES and the T/S ratio; clinical covariables were intentionally excluded from the clustering to focus purely on biological phenotypes. We fitted a series of LCA models starting from *k* = 1 (where k is the number of classes) onward. The optimal value of k was determined using model-fit indices, primarily the Bayesian Information Criterion (BIC), for which lower values indicate better model fit. The Akaike Information Criterion (AIC) was also considered. Model classification certainty was assessed via Mean Posterior Probabilities (MPP) [17]. and Normalized Entropy. An MPP>0.70 was considered indicative of robust individual classification. To ensure clinical interpretability, we ensured that the smallest class size encompassed ≥1.5 % of the entire cohort. It should be noticed that such assignment is deterministic and ignores the probabilistic uncertainty of class membership. Even though more advanced approaches, such as the Bolck-Croon-Hagenaars (BCH) method, exist to incorporate this uncertainty, the three-step approach remains robustly supported in applied health research, especially when the final model demonstrates high individual classification certainty.

**Step 2** **: Prognostic evaluation: outcome and Survival Analysis**

The latent classes defined in Step 1 were subsequently used as the primary predictors in the outcome analysis, following a standard, robust three-step procedure common in latent variable modeling: Step 1: Estimation of the Latent Class Model (LCA); Step 2: Assignment of individuals to their Most Likely Class (MLC) based on their highest posterior probability; Step 3: Regression of the outcome on the assigned class using traditional regression (Cox/Fine & Gray). Survival analyses for Overall Survival (OS) were performed using Kaplan–Meier estimates with the log-rank test. The effect of the latent classes was further evaluated using a multivariate Cox Proportional Hazards model (built on the entire cohort), adjusting for chronological age. The details of the Cox models, including Hazard Ratios (HR), 95 % Confidence Intervals (CI), and p-values, will be fully reported in the Results section. To address the risk of confounding factors and multiple testing: (1) the proportional Hazards assumption for the final Cox model was formally tested using the Schoenfeld residuals (via the `cox.zph` function). If the assumption was violated, the model would be adjusted using time-dependent covariates or stratified Cox regression; (2) a competing risk analysis (CRA) was performed using the Fine and Gray subdistribution hazard model, given the nature of OS in an older oncological cohort (risk of cancer death vs. non-cancer death), contingent on the findings of the primary Cox model and (3) considering the impact of individual biomarkers on OS, the risk of false positives due to multiple testing was controlled by reporting the False Discovery Rate (FDR) adjusted p-values (q-values).

**Step 3** **: Structural Equation Modeling and Clinical Feasibility Validation**

The objective of this step was to develop a tool that is easily transferable to routine clinical practice. To visualize the statistical associations between the identified set of biomarkers and the patient outcomes, we employed a multi-step analytical strategy. Following the identification of latent classes via Latent Class Analysis (LCA), Structural Equation Modeling (SEM) was conducted using the latent class assignment as the primary categorical endogenous variable (outcome). The primary objectives of the SEM were two-fold: (1) to visually highlight the multivariate statistical associations between the input biomarkers and the latent class membership, and (2) to identify the minimal set of biomarkers that best predicted latent class assignment, thereby creating a feasible predictive model for future clinical applications. Crucially, in adherence to the established methodological limitations of standard SEM concerning censored data, the structural model was restricted to non-censored outcomes. Specifically, the SEM assessed the relationship between the biomarkers and the categorical latent class assignment, and separately, the binary vital status (deceased/alive) as a dichotomous outcome. The SEM was not used to model the time-to-event variable. We then performed binary logistic regression to predict membership into the high-risk classes (combined classes) using a minimal sub-set of biomarkers. It is critical to emphasize that this step is a binary classification analysis and not a survival analysis. The performance of this classification model in predicting latent class membership was evaluated by the Area Under the Curve (AUC) of the Receiver Operating Characteristic (ROC). To evaluate the robustness of this high discriminative capacity and account for potential overfitting, we performed internal validation using the Bootstrap method (*B* = 1000 resamples). This procedure provided an estimate of the optimism bias and allowed for the calculation of a corrected (unbiased) AUC with its 95 % Confidence Interval.

All tests were two-sided, and the threshold for statistical significance was set to *p* < 0.05. All statistical analyses were performed using R software version 4.3.0 (The R Foundation for Statistical Computing, Vienna, Austria). URL http://www.R-project.org/).

## Results

### Population characteristics

The patients were recruited between March 2004, and July 2011; the median follow-up was 129 months. The main patient and tumor characteristics of the “Old” and “Young” groups of patients are presented in Table 1.

**Table 1:** Characteristics of the “Old” and “Young” groups of patients at inclusion and during their follow-up.

	“Old” group *N* = 162	“Young” group *N* = 82
Age at diagnosis (median [IQR])	76.0 [72.0 - 80.0]	40.0 [37.0 - 44.0]
Tumor histological subtype ( %) IBC—NST ILC Other	*N* = 162 68.5 15.4 16.0	*N* = 82 90.2 9.8 0
Surrogate Molecular subtype ( %) Luminal A Luminal B Luminal B/Her2-positive Her2-positive Triple negative	*N* = 162 61.1 17.9 7.4 4.9 9.8	*N* = 82 59.8 18.3 7.3 4.9 8.6
Tumor grade ( %) 1 2 3 Unknown	*N* = 162 15.4 47.5 36.4 0.6	*N* = 82 14.6 47.6 37.8 0
Neoadjuvant treatment ( %)	11.1	9.8
Tumor size pT1 pT2 pT3 pT4 pTx	*N* = 144 28.1 54.9 5.6 1.4 0	*N* = 74 47.3 44.6 5.4 1.4 1.4
Lymph node involvement pN0 pN1 pN2 pN3 pNx	*N* = 144 57.6 29.2 6.9 5.6 0.7	*N* = 74 59.5 20.3 12.2 8.1 0
Median follow up (months)	107.17	165.68
Deaths ( %)	64.20	13.41

Abbreviations: IBC—NST: invasive breast carcinoma of no special type; ILC: invasive lobular carcinoma.

### Sensitivity analysis and justification of primary model

The choice of Multiple Imputation by Chained Equations (MICE) as the primary analytical strategy, under the assumption of Missing At Random (MAR) data, was validated through a comprehensive sensitivity analysis comparing its pooled results to the Complete Case Analysis (CCA). This comparison demonstrated that CCA introduced significant selection bias and loss of statistical power. Since MICE utilizes all available data, thereby providing more robust and efficient estimates compared to the biased subset used in CCA, these results confirm the methodological superiority of imputation in this context, supporting the final conclusions drawn from the MICE-pooled results.

### Correlation matrix of the biological variables

**Fig. 1:**
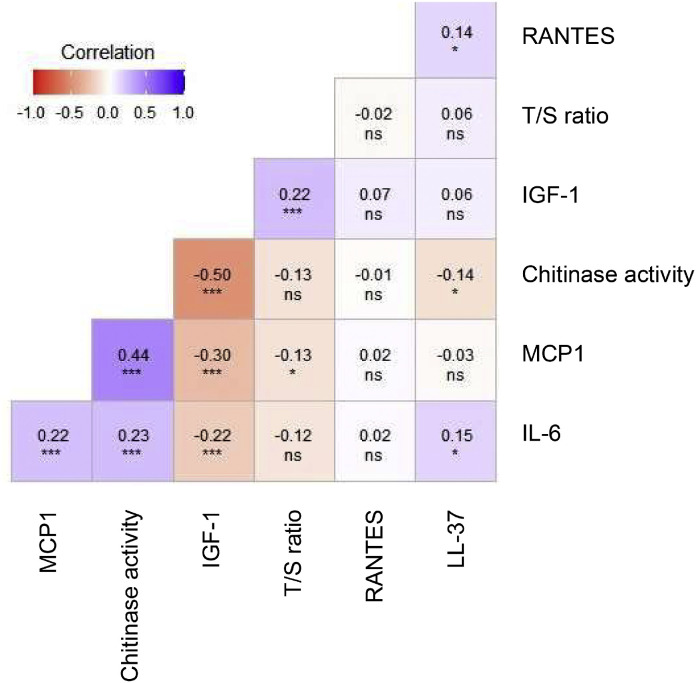
Spearman correlation matrix for tested biological values (ns: *p* ≥ 0.05; *: *p* < 0.05; **: *p* < 0.01; ***: *p* < 0.001).

The correlation matrix of the tested biomarkers is presented in Fig. 1. LL-37 was mildly positively correlated with RANTES (ρ = 0.14) and IL6 (ρ = 0.15) and negatively correlated with chitinase activity (ρ = - 0.14, all *p* < 0.05). IGF-1 was strongly positively correlated with the T/S ratio (ρ = 0.22) and negatively correlated with IL6 (ρ = -0.22), MCP1 (ρ = -0.30) and chitinase activity (ρ = -0.50) (all *p* < 0.001). Chitinase activity was strongly positively correlated with IL6 (ρ = 0.23) and MCP1 (ρ = 0.44); MCP1 and IL6 were also strongly and positively correlated (ρ = -0.22, all *p* < 0.001).

### Associations between aging biomarkers and OS

**Fig. 2:**
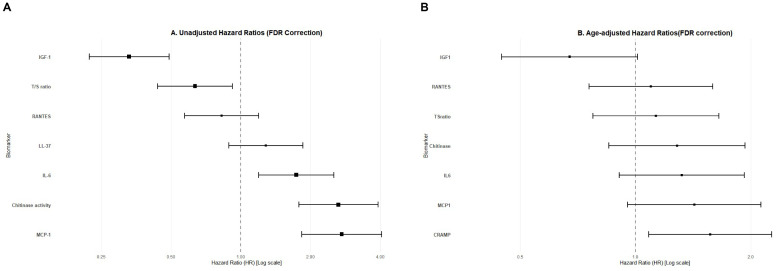
Univariate (A) and age-adjusted (B) associations of each biomarker with overall survival. . Biomarkers retaining significance using False Discovery Rate (FDR) adjusted p-values (q-values < 0.05) are presented as big squares.

At the cutoff date, 115 of the 244 patients had died, and 29 (25.2 %) deaths were cancer related. Among those in the “Old” group, 104 patients (64.2 %) died, and 20 deaths (19.2 %) were related to cancer. Considering the “young” group, 11 deaths were recorded (13.4 %), of which 9 (81.8 %) were cancer related. To evaluate the associations between aging biomarkers and overall survival, continuous variables were transformed into 2-class categorical variables, and the corresponding median value was used as the cutoff limit. The univariate and age-adjusted associations of each biomarker with OS were analyzed via the log-rank method in the whole cohort (Fig. 2). Univariate analysis revealed that high chitinase (HR: 2.64 [95 % CI: 1.78–3.92]) high IL6 (HR: 1.74 [95 % CI: 1.20–2.53]),and high MCP-1 (HR: 2.73 [95 % CI: 1.84–2.06]) levels were associated with poor survival, whereas high IGF-1 (HR: 2.73 [95 % CI: 1.84–2.06]) levels appeared to be protective. After age adjustment, only high LL-37 (HR=1.57 [95 % CI: 1.08–2.27]) conferred a negative outcome. Finally, and after False Discovery Rate (FDR) adjustment (q-values), none of the variables retained significance, confirming the need for a combined, multivariate approach to risk stratification. Additionally, we performed a multivariate analysis using log-transformed continuous values of MCP-1, Chitinase, and IGF-1, which failed to show any statistically significant association with OS (all *P* > 0.05). These results further indicate that the prognostic value of these biomarkers is threshold-dependent and synergistic rather than linear, justifying the use of a latent class-based categorization.

### Latent class analysis

Considering the interconnection of some of the biomarkers according to common aging-related pathways and their expected statistical interaction, we decided to perform a latent class analysis on the whole population cohort to identify distinct biologic profile(s). A model selection process considering models with *K* = 1 to *K* = 5 classes was performed. The two-class model (*K* = 2) was ultimately selected based on three critical criteria: (1) Model parsimony: the *K* = 2 model yielded the lowest Bayesian Information Criterion (BIC = 2356.00) and AIC (2303.55), indicating the best fit for the data, (2) Superior robustness and convergence: Models with *K* = 3 and *K* = 5 classes failed to reach convergence during the estimation process (maximum likelihood not found alert), which compromised the reliability of their parameter estimates and precluded their selection. In contrast, the *K* = 2 model demonstrated full convergence without warnings. Prior to adopting this categorical approach, we systematically attempted to model the biomarkers as continuous variables using Latent Profile Analysis (LPA). However, these attempts proved unsuccessful, as the multivariate distribution of the biomarkers significantly deviated from normality (Mardia’s test for skewness, *p* < 0.001). Furthermore, all tested LPA models—ranging from highly flexible to strictly constrained—either failed to reach numerical convergence or resulted in highly unstable solutions with clinically non-viable class sizes (e.g., 1.2 % of the cohort). and (3) Classification certainty and clinical interpretability: the final *K* = 2 model demonstrated high individual classification certainty, with a Mean Posterior Probability (MPP) of 0.8905, well above the standard 0.70 threshold. While the normalized entropy was moderate (0.6213), reflecting the expected biological overlap in clinical phenotypes, the high MPP confirms that patients were assigned to their respective classes with a high degree of confidence (89 % on average). The model yielded two distinct, clinically meaningful profiles. The log-likelihood of the final model was −1136.77. The smallest resulting class (Class 2, *N* = 107) represented approximately 43 % of the cohort, satisfying the minimum size criterion of 1.5 %. The clinical and biological characteristics of each class in this 2-class model are presented in Table 2.

**Table 2:** Characteristics of the two latent classes.

	Total	Class 1	Class 2
Age (years) Range Median [Q1-Q3] Mean	*N* = 244 27 – 90 72 [44 – 78.25] 64.48	*N* = 137 35 – 90 76 [71 – 81] 73.97	*N* = 107 27 – 90 44 [38 – 72.50] 52.33
Postmenopausal status (proportion)	*N* = 244 68.39 %	*N* = 137 91.24 %	*N* = 105 38.57 %
T/S ratio Range Median [Q1-Q3] Mean	*N* = 196 0.51 – 2.19 0.86 [0.70 – 0.99] 0.88	*N* = 101 0.51 – 1.94 0.76 [0.65 – 0.95] 0.82	*N* = 95 0.55 – 2.19 0.93 [0.81 – 1.04] 0.96
LL-37 (ng/mL) Range Median [Q1-Q3] Mean	*N* = 124 16.89 – 179.38 45.61 [32.24 – 56.21] 47.49	*N* = 43 16.89 – 179.38 45.60 [31.02 – 56.62] 47.31	*N* = 81 19.73 – 92.58 45.62 [34.07 – 55.75] 47.59
Chitinase activity (U/mL) Range Median [Q1-Q3] Mean	*N* = 123 127.00 – 3051.40 1092.90 [963.10 – 1522.40] 1257.20	*N* = 43 970.80 – 3051.40 1594.50 [1407.90 – 1875.50] 1673.40	*N* = 80 127.00 – 1904.30 1037.60 [904.40 – 1124.30] 1033.50
RANTES (ng/mL) Range Median [Q1-Q3] Mean	*N* = 238 1.72 – 208.13 78.17 [59.19 – 100.08] 81.38	*N* = 133 1.72 – 208.13 79.82 [59.21 – 103.76] 84.02	*N* = 105 5.76 – 177.66 77.55 [59.18 – 96.65] 78.03
IL6 (pg/mL) Range Median [Q1-Q3] Mean	*N* = 238 0.00 – 277.98 1.75 [0.34 – 3.41] 5.87	*N* = 133 0.00 – 92.51 2.70 [0.95 – 4.99] 6.11	*N* = 105 0.00 – 277.98 0.83 [0 – 2.33] 5.58
MCP1 (pg/mL) Range Median [Q1-Q3] Mean	*N* = 238 17.10 – 2296.00 112.90 [80.95 – 164.00] 137.36	*N* = 133 41.50 – 2296.00 141.30 [110.90- 197.20] 175.40	*N* = 105 17.10 – 217.50 79.70 [67 – 105.80] 89.18
IGF-1 (pg/mL) Range Median [Q1-Q3] Mean	*N* = 213 27.60 – 249.70 79.80 [63.10 – 106.00] 85.65	*N* = 116 27.60 – 195.90 65.40 [52.92 – 76.15] 69.14	*N* = 97 42.80 – 249.70 98.80 [88.80 – 120.80] 105.4

In brief, patients in class 1 tended to be older, more frequently postmenopausal, have a lower mean leukocyte telomere length (T/S ratio), higher serum chitinase activity, higher serum levels of IL6, RANTES, and MCP1, and a lower level of IGF1.

### Relationships between latent classes, biomarkers and aging

**Fig. 3:**
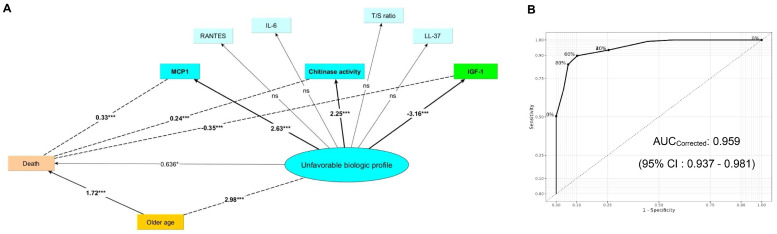
Structural equation model of the relationships between latent classes, age, aging biomarkers. LCA of the whole biomarker dataset identified 2 latent classes (1: 137 pts; 2: 107 pts). Class 1 corresponded to an unfavorable biologic profile significantly related to high MCP1, high chitinase activity and low IGF-1. A: Schematic diagram of the LCA; Circle = latent variable; Rectangles = observed variables (blue: unfavorable biomarkers; green: favorable biomarkers; orange: outcomes; yellow: clinical variables); Univariate regression models: dotted lines; Multivariate regression models: solid lines; p value: *< 0.05, ***<0.001. B: ROC curve of the model using MCP1, chitinase activity and IGF-1 as predictors.

A structural equation model was developed to represent (dotted lines) the direct and indirect effects of latent variables on OS (biomarkers or clinical variables), presented as standardized (correlation) coefficients (Fig. 3A, dotted lines). A multivariate logistic regression analysis was performed to represent the biomarkers significantly predicting class assignment and their standardized (β) coefficients: the class 1 biological profile was significantly positively related to the MCP1 level (β: 2.63, *p* < 0.001), chitinase activity (β: 2.25, *p* < 0.001), and negatively related to the IGF1 level (β: −3.16, *p* < 0.001) (Fig. 3A, solid lines). The discriminative performance of the logistic regression model was assessed using the internal validation bootstrap method (***B*** = 1000 samples). The initial Area Under the Curve (AUC), measured on the training dataset, was 0.964. The calculated mean optimism (overfitting) was found to be minimal (0.005), resulting in a corrected (unbiased) AUC of 0.959 (95 % CI 0.937–0.981). This value confirms the model's robustness and generalizability for differentiating between the two latent classes." (Fig. 3B). To evaluate the relationships between latent class 1 and patients’ clinical characteristics and outcomes, univariate regression analyses were performed between assignment to latent class 1, age and the risk of premature death. As presented, a significant association was demonstrated, leading to the renaming of latent class 1 to the “unfavorable biologic profile” (Fig. 3A, solid lines) in contrast to class 2 or the “favorable risk profile”.

### Class 1 assignment according to age groups and survival analyses

**Fig. 4:**
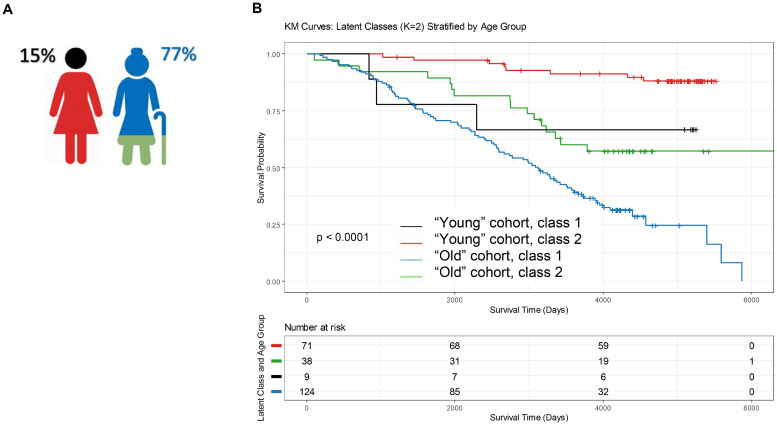
A stratified analysis of survival according to the “young” and “old” groups of patients. A: Schematic representation of the proportions of the subgroup with unfavorable biologic profiles in the “young” and “old” groups of patients. B: Kaplan‒Meier estimates of overall survival according to age groups and latent classes (1= unfavorable biologic profile versus 2).

After adjustment on age (longitudinal) class 1 assignment was strongly associated with poorer OS (Age-adjusted HR=1.82 [95 %CI 1.11–2.99], *p* = 0.018). The primary multivariate Cox Proportional Hazards Model showed no violation of the assumption, with the global p-value for the Schoenfeld residuals being *p* = 0.71. This confirms the validity of the hazard proportionality for the latent class variable. Considering that the unfavorable biologic profile is significantly related to age but may also concern younger patients and with the repartition structure of the cohort, class 1 and 2 assignments were analyzed in the different age groups. As presented in Fig. 4A, the unfavorable biologic profile (class 1) represented 77 % of the Old group and 15 % of the Young group. After adjustment for age groups (HR=5.41 for the “Old” age group versus the “Young” age group, [95 % CI: 2.70–10.86], *p* < 0.001), unfavorable biological profile (class 1) assignment was associated with significantly poorer survival (HR=1.66, [95 % CI: 1.03–2.68], *p* = 0.039) (Fig. 4B). The Kaplan–Meier curves (Fig. 4B) visually confirmed the strong, four-way risk stratification resulting from the combination of age and biologic profile. The combination of the Unfavorable Biologic Profile (Class 1) and Older Age resulted in the poorest overall survival, while the Favorable Risk Profile (Class 2) in Younger Patients maintained the highest survival rate over the entire follow-up period.

A subsequent Competing Risks Analysis (CRA**)** using the Fine and Gray model was performed to distinguish between cancer-specific death and other causes of death: (1) Cancer-Specific Death (CSD): assignment to the unfavorable biologic profile (Class 1) was more strongly associated with CSD than with OS (Subdistribution HR: 2.05 [95 % CI: 1.10–4.20], *p* = 0.012), consistent with a statistical association between the unfavorable biologic profile and cancer aggressiveness (2) Non-Cancer Death (NCD): Assignment to the unfavorable biologic profile (Class 1) showed a strong trend towards increased NCD risk (Subdistribution HR: 1.45 [95 % CI: 0.95–2.20], *p* = 0.09), consistent with the profile's strong correlation with advanced age and general frailty.

## Discussion

In this study, we applied Latent Class Analysis (LCA) to an integrated panel of biomarkers related to aging and metabolic health in a large cohort of breast cancer patients, successfully identifying two distinct biological phenotypes: a Favorable Risk Profile (Class 1) and an Unfavorable Biologic Profile (Class 2). Our primary finding is that assignment to the Unfavorable Biologic Profile is a powerful, independent predictor of poor Overall Survival, with prognostic capacity that significantly exceeds that of chronological age alone. This demonstrates that an objective biological aging signature is a more critical determinant of outcome than a patient's years lived, fundamentally supporting the concept of biological heterogeneity in geriatric oncology.

The Unfavorable Profile was characterized by high levels of Monocyte Chemoattractant Protein-1 (MCP-1), high Chitinase activity, and low Insulin-like Growth Factor-1 (IGF-1). The elevated MCP-1 is a key finding, as this chemokine is a potent attractant for monocytes and macrophages, driving a pro-inflammatory microenvironment. While often studied in the context of tumor recruitment, our finding that high MCP-1 remained significantly associated with poor survival independent of tumor characteristics, clinical stage, and treatment type, suggests that it is more a marker of systemic vulnerability and biological frailty than a tumor-specific prognosticator.

The unfavorable profile also showed high Chitinase activity and low IGF-1 levels. Chitinase activity, particularly chitotriosidase, is strongly associated with macrophage activation, lysosomal dysfunction, and chronic inflammation. Its concurrent elevation with MCP-1 reinforces the concept that the "unfavorable biologic profile" represents a state of high systemic inflammaging—a chronic, sterile, low-grade inflammation that is a key driver of aging and age-related disease [18]. The simultaneous finding of low IGF-1 completes this picture of systemic distress. IGF-1 is central to the nutrient-sensing pathway and serves as a powerful anabolic signal. Reduced IGF-1 levels are frequently observed in states of catabolism, malnutrition, and frailty (sarcopenia), reflecting a collapse of the anabolic reserve that is critical for host resilience and tissue repair [19]. The triade of high inflammatory signals (MCP-1, Chitinase) and a compromised anabolic state (low IGF-1) defines a patient phenotype with critically low homeostatic reserve, consistent with the definition of pathological aging.

Crucially, the competing risks analysis (CRA) provided a critical answer to our initial biological question regarding the host-tumor interplay. We observed that assignment to the unfavorable profile was more strongly associated with Cancer-Specific Death (CSD) than with Non-Cancer Death (NCD). This suggests that the hostile "terrain" captured by the profile—characterized by chronic inflammation and low anabolic reserve—does not simply increase the risk of dying from *any* cause. Instead, it seems to fundamentally impair the host's ability to tolerate, manage, or respond effectively to the malignancy and its associated treatments, ultimately enhancing the lethality of the cancer itself. This supports the hypothesis that a pre-existing systemic biological vulnerability promotes the tumor's aggressiveness or limits treatment efficacy.

Beyond the survival outcome, the LCA successfully identified a significant proportion (15 %) of patients in the chronologically "Young" cohort who were assigned to the unfavorable profile. Conversely, 23 % of the "Old" cohort were assigned to the favorable profile (Class 2). This confirms that chronological age is a poor proxy for biological risk and validates the utility of the LCA-derived profiles as objective metrics of biological age and host heterogeneity, independent of years lived. The resulting four-way risk stratification (Age x Profile) confirms that the biological profile provides significant prognostic information beyond chronological age alone.

The objective identification of these latent biological profiles offers direct translational relevance, which is essential for refining individualized treatment strategies in oncology. The major clinical utility of our findings resides in the fact that the latent class profile, derived from a minimal set of easily measurable and reproducible blood-based biomarkers (MCP-1, Chitinase activity, and IGF-1), provides a rapid, objective, and scalable screening tool to identify high-risk patients. This composite biomarker can serve as a robust pre-screening mechanism within the breast center, enabling clinicians to target biologically frail patients (those in the unfavorable profile) before major therapeutic decisions are made. The identification of such a profile should logically trigger a necessary Comprehensive Geriatric Assessment (CGA) and intensive supportive care interventions (such as nutritional support or prehabilitation).

Furthermore, given the strong link between the unfavorable profile and Cancer-Specific Death (CSD), this objective signature validates the need for a critical review of clinical decisions. It supports a biomarker-guided approach for therapeutic de-escalation (e.g., favoring endocrine therapy over adjuvant chemotherapy) in older or vulnerable patients, with the aim of maximizing quality of life and potentially improving long-term survival by avoiding cumulative toxicity. Finally, the identified unfavorable biological profile may be considered a target for future intervention studies. These studies could take the form of lifestyle interventions, known to reduce chronic inflammation and activate anabolic pathways [20], or be part of the context of anti-aging therapies, such as senolytics or senomorphics.

In conclusion, by applying Latent Class Analysis to an integrated panel of biological aging markers in a breast cancer cohort, we successfully identified two distinct patient profiles: a Favorable Risk Profile and an Unfavorable Biologic Profile characterized by a triade of high MCP-1 and Chitinase activity, and low IGF-1 levels. This unfavorable profile is a powerful independent predictor of poor Overall Survival, and notably, it is strongly and specifically associated with Cancer-Specific Death, demonstrating that the host's underlying biological age, rather than merely chronological age, plays a critical role in the lethality of breast cancer. These findings provide an objective, translatable, and integrated biomarker signature for stratifying risk and guiding treatment intensity in geriatric oncology.

## Ethics approval and consent to participate

All clinical data were collected in compliance with the Helsinki Declaration. Blood sampling, collection of patient data, and genetic analysis were approved by the ethics committee of University Hospitals Leuven (Trial registration: BS32220096117). All patients included in the study provided written informed consent.

## Consent for publication

All authors of the manuscript have read and agreed to its content and are accountable for all aspects of the accuracy and integrity of the manuscript in accordance with ICMJE criteria**.**

## Availability of data and materials

Currently, no mechanism is in place to allow the sharing of individual deidentified patient data. Requests sent to the corresponding author are considered on a case-by-case basis.

## Funding

CF reported a grant from La Ligue Nationale contre le Cancer for funding for the research; BB reported a grant from Vlaamse Liga tegen Kanker (VLK) for the conduct of the study; HW reported a grant from the Fund for Scientific Research Flanders (FWO Vlaanderen).

## CRediT authorship contribution statement

**Claire Falandry:** Writing – review & editing, Writing – original draft, Visualization, Validation, Supervision, Software, Resources, Project administration, Methodology, Investigation, Funding acquisition, Formal analysis, Data curation, Conceptualization. **Sigrid Hatse:** Writing – review & editing, Visualization, Validation, Project administration, Investigation, Data curation, Conceptualization. **Barbara Brouwers:** Writing – review & editing, Writing – original draft, Visualization, Validation, Supervision, Resources, Project administration, Methodology, Investigation, Funding acquisition, Formal analysis, Data curation, Conceptualization. **Cindy Kenis:** Writing – review & editing, Visualization, Validation, Project administration, Investigation, Data curation. **Ann Smeets:** Writing – review & editing, Visualization, Validation, Investigation. **Patrick Neven:** Writing – review & editing, Visualization, Validation, Investigation. **Charlotte Cuerq:** Writing – review & editing, Visualization, Validation, Investigation. **Frederic Pamoukdjian:** Writing – review & editing, Visualization, Validation, Software. **Karim Chikh:** Writing – review & editing, Visualization, Validation, Software, Investigation, Conceptualization. **Hans Wildiers:** Writing – review & editing, Writing – original draft, Visualization, Validation, Supervision, Software, Resources, Project administration, Methodology, Investigation, Funding acquisition, Formal analysis, Data curation, Conceptualization.

## Declaration of competing interest

The authors declare that they have no known competing financial interests or personal relationships that could have appeared to influence the work reported in this paper.

## Appendix Supplementary materials

Image, application 1

Image, application 2

Image, application 3

Image, application 4
